# Impact of the COVID-19 pandemic on digestive cancer staging, a case series

**DOI:** 10.1016/j.amsu.2022.104471

**Published:** 2022-08-27

**Authors:** Omar Mouni, Anass Idrissi, Mohamed Bouziane, Samir Ahid, Khalid Sair

**Affiliations:** a Faculty of Medicine, Mohammed VI University of Health Sciences (UM6SS), Casablanca, Morocco; b Methodological Support Unit, Faculty of Pharmacy, Mohammed VI University of Health Science, Casablanca, Morocco

**Keywords:** COVID-19, Digestive cancer, Staging

## Abstract

**Introduction:**

The COVID-19 pandemic had an undeniable impact on the health system worldwide, this lead to a delay in the diagnosis and treatment of digestive cancers.

The purpose of our study was to assess this delay and its impact on patient care.

**Method:**

Our work is a retrospective study about 165 patients that were admitted for digestive cancers at Sheik Khalifa hospital, Casablanca morocco during a 3-year period, that we divided into three. We included all the digestive adenocarcinomas (esophagus excluded) whether they were operated on or not. We excluded all other types of cancers (GIST, serous tumors …). We assessed the time between the beginning of the symptoms and the beginning of the treatment and the number of patients that were diagnosed at the complication stage. We also assessed the staging of the tumor at the moment of diagnosis and the complete surgical resection rate.

**Results:**

Among the 165 patients admitted for digestive cancer, 54,9% were males with a sex ratio of 1,22 M/F. The average age of our patients was 62,8 years varying between 25 and 86 years old and with a standard deviation of 11,8 years. Digestive cancers were diagnosed in 79 patients during period 1, 43 patients during period2, and 43 during period 3. We found a statistically significant increase in the percentage of patients with advanced cancer by 21,7% (p = 0,045) from 2019 to 2020. The delay in diagnosis (p = 0,275), percentage of cancer discovered at the stage of complication(p = 0,728), and the reduction in complete surgical resection (p = 0,177) were not statistically significant.

**Conclusion:**

Our results show an undeniable impact of the COVID-19 pandemic on the staging of digestive cancers but the impact on their care remains to be proven and needs a long-term survival follow-up.

## Highlights

• This is study assesses the impact of The COVID-19 pandemic on the diagnosis delay in digestive cancers and its impact on their staging.
• A three-year study that includes 165 patients, 1 year before the COVID-19 pandemic and two years during.
• The pandemic had a statistically significant impact on the staging of the tumor but no impact on the short-term outcome of the patients.
• The first study of its kind in Morocco.

## 1 Introduction

The COVID-19 pandemic, as in anywhere else in the world, had an undeniable effect on the health system in morocco [[1], [2], [3]]. Many diseases have known a certain delay in diagnosis and in their treatment including digestive cancers. There has been an estimated 2.3 million cancer operation canceled during the height of the pandemic [4,5].

In March 2020, the Moroccan government declared a state of public health emergency and placed many restrictions in order to limit the spread of the virus (permits to leave home, permits to travel between cities …) [6] this has severely limited access to health facilities and caused a delay in cancer screening and diagnosis. There was also a reluctance of patients to seek medical help due to a fear of infection or overburdening the healthcare system.

Patients with cancer were still being treated, but the care provided was severely impacted, as many resources were diverted in order to respond to the pandemic.

Early diagnosis and treatment have a major impact on the prognosis of any cancer [7,8] and any delay may lead to a progression of the disease and can influence directly the final outcome of the patient. This may have caused an advancement of the stage of the disease or impacted the treatment outcome as some cancers may have become metastatic or inoperable during this delay. More acute presentation may have appeared such as digestive bleeding or occlusion and this has a direct impact on the prognosis of the disease.

In morocco there was no official unified guideline for the care of patients with digestive cancer or cancer in general during the pandemic, also the COVID-19 pandemic impacted each region differently as the Casablanca region was most heavily impacted [6], this led to disparate care provided for cancer patients that varies amongst regions and even hospitals and may cause different long term impact of the pandemic for each region.

Many studies have evaluated the impact of the COVID-19 pandemic on digestive and breast cancer screening, presentation, and treatment [[9], [10], [11], [12]] and on the impact of the COVID-19 pandemic on cancer deaths in general [13] but our study is the first of its kind in morocco relating to digestive cancers, The aim of our study was to determine whether the eventual delay in diagnosis had any impact on the staging of digestive cancers and on the quality of care provided for our patients. it can help us learn a vital lesson for the future, and help us avoid any mistakes that were made, especially in the context of the Sars-CoV-2 variants and the subsequent waves of the pandemic that may occur.

## 2 Methods

This is a retrospective study that included 165 patients admitted to Sheik Khalifa university Hospital in Casablanca, Morocco for adenocarcinoma of the stomach, pancreas, colon, and rectum between February 2019 to September 2021. Almost three years that we divided into three periods: period 1: from February 2019 to January 2020; period 2: from February 2020 to January 2021 (first year of the COVID-19 pandemic); period 3: from February 2021 to September 2021 (nine months).

We excluded all other types of carcinomas (GIST, serous tumors …) because of the differences in classifications. We also excluded all esophageal tumors due to the lack of necessity for a surgical excision in most cases.

For the patients that underwent radiotherapy before surgery, we opted for the radiological staging rather than the pathology report, because it reflects better the real stage of the disease (regression under treatment even a possible disappearance of the tumor).

For each patient, we gathered: the age, the sex, the location of the cancer, the time between the beginning of the symptoms and the beginning of the treatment (surgery or radiotherapy), whether or not the cancer was discovered at the complication stage (complete digestive occlusion, digestive bleeding, or tumor perforation), the staging of the tumor on the pathology study of the surgical specimen when surgical resection was done and on the radiological report when surgery was deemed impossible or the patient received neoadjuvant treatment. our staging was based on the TNM classification in its latest update [[14], [15], [16], [17], [18], [19], [20]], and finally whether or not the tumor was completely removed.

The surgery was performed by a team of surgeons with experience ranging between 30 and 10 years in digestive and endoscopic surgery. The patients underwent rigorous pre-operative preparation, especially from a nutritional standpoint. Patients who had cancers of the left colon or rectum also received a mechanical bowel preparation.

The data were collected from medical records. The data analysis was performed using Chi-square and Kruskal-Wallis nonparametric one-way analysis of variance on the JAMOVI software version 2.3.12.0. A p-value of <0.05 was considered statistically significant.

This work has been reported in line with the PROCESS 2020 (www.processguideline.com) [21].

## 3 Results

The mean age of our patients was 62,8 years with a standard deviation of 11.8 years, varying between 25 and 86 years old. 54,9% were males with a sex ratio of 1,22 M/F. Digestive cancers were diagnosed in 79 patients during period 1, 43 patients during period2, and 43 during period 3. We included all the patients diagnosed with stomach, pancreas, colon, or rectal adenocarcinomas (Table 1) (see Table 2).

**Table 1:** Location of the cancer.

	period			
**Localization**	1 N(%)	2 N(%)	3 N(%)	Total N(%)
**Stomach**	5 (6.3)	6 (14)	6(14)	17(10.3)
**Pancreas**	11(13.9)	4 (9.3)	3 (7)	18(10.9)
**Right colon**	10 (12.7)	10(23.7)	4(9.3)	24 (14.5)
**Left colon**	29 (36.7)	11 (25.6)	16 (37.2)	56 (33.9)
**Rectum**	24 (30.4)	12 (27.9)	14 (32.6)	50 (30.3)
**Total**	79 (100)	43(100)	43(100)	165 (100)

**Table 2:** Summarizing the results of our study.

	period			p-value
	1	2	3	
Time between the beginning of symptoms and treatment<u>^a^</u>	17.8 [11; 22]	20.0 [11; 22]	18.2 [11; 18.5]	0,275
Cancers discovered at the stage of complication (%)	21.5	27.9	23.3	0,728
Cancers discovered at an advanced stage (stage 3–4) (%)	62.0	83.7	69.8	**0,045**
Complete surgical resection(%)	78.5	62.8	72.1	0,177

a Time in weeks and quartiles.

The median time between the beginning of symptoms and treatment (surgery or neo-adjuvant treatment) was 18,5 weeks [11; 23]. There were no statistically significant differences in the median delay of treatment between each period (p = 0,275).

We also compared the number of cancers diagnosed at the complication stage (complete digestive occlusion, digestive bleeding, or tumor perforation). We found no difference statistically significant in the number of patients diagnosed at the complication stage of the disease each period (p = 0,728).

We compared the number of patients that were diagnosed at an early stage of the disease (stage 1–2) versus the number of patients that were diagnosed at a more advanced stage (stage 3–4) for each period. We found that the percentage of diagnoses at an advanced stage went up from 62% to 83,7% during the second period only to go down to 69,8% during the third period (p = 0,045).

We also compared the number of patients that underwent surgery with complete resection of the tumor. We found no statistically significant difference in the percentage of complete surgical resection between each period (p = 0,177).

## 4 Discussion

The COVID-19 pandemic, as in anywhere else in the world, had an undeniable effect on the health system in morocco. In march 2020 the Moroccan government declared a state of public health emergency and placed many restrictions in order to limit the spread of the virus (permits to leave home, permits to travel between cities …) [6] these restrictions caused a delay in the consultations for many patients causing an automatic delay in diagnosis which was done at a more advanced stage of the disease [22].

Our study is the first of its kind in morocco concerning digestive cancers, other studies have evaluated the impact of the COVID-19 pandemic on digestive and breast cancer screening, presentation, and treatment [[9], [10], [11], [12]].

In our study, we found no significant delay in diagnosis as reported by S. Aguiar Jr and al [9] and we found no change in the percentage of cancer diagnosed at the complication stage as found by Jian Cui and al [23] who also found a decrease in the number of asymptomatic cases this number is already extremely low in our country, as there is no national screening plan for digestive cancers [[24], [25]].

In our study, the percentage of advanced cancers went up by 21,7% from the first to the second period only to go down by 13,9% in the third. Other studies about the effect of delayed diagnosis on cancer staging found similar results if the delay of diagnosis is more than a year [7]. The decrease of this percentage in the last period suggests a possible return to normal.

Where digestive cancers were concerned we did not need to delay any diagnostic or therapeutic procedure in our hospital as the COVID-19 pandemic was relatively kept under control compared to other countries especially in Europe [14,26]. Our hospital was never completely at overcapacity so we opted to keep our procedure as usual so as to give our patients as much of a survival chance as possible.

Nevertheless, we found a significant reduction in the number of diagnosed cases: from 79 to 43 during the same period of the following year (a reduction of 54%); the reduction was reported in other studies [[9], [10], [11], [12]].

This reduction can be explained by the fact that in our country as in many others there was a significant decrease of patients that underwent routine physical examination; to avoid long trips and because of travel restrictions, patients with mild clinical symptoms chose a community hospital or nearby hospital or received treatment in their residence (without further examination) a lot of tertiary hospitals gave priority to critically ill patients [23,27,28]; this led to a reduction in the number of patients that were referred to our hospital. In period 3 we found a slight resurgence of cases: 43 cases in a period of only 9 months, which may suggest a return to a regular situation.

The complete surgical resection of the cancer was our treatment of choice when possible, with adjuvant or neoadjuvant chemo or radiotherapy when necessary. We followed the classical recommendation for digestive surgery [[14], [15], [16], [17], [18], [19], [20]] and opted out of the new French recommendation for the COVID-19 pandemic [29] that suggested the delay of surgery in favor of adjuvant treatment, because we deemed them unjustified, as our hospital was never in over capacity even during the peak of the pandemic.

The increase in advanced cases did not have an immediate impact on the possibility of complete surgical resection and in theory, should not have any as it was demonstrated in other studies that a delay in diagnosis does not have any effect on the long-term survival rate if that delay does not exceed 90 days [7,30,31], another study in Denmark found that there was no short term impact of the pandemic on the care of patients with colorectal cancer [11] but the increase in advanced case we found may suggest otherwise and its effect on long term survival remain to be studied as there has been indeed a predicted increase of cancer death due to the COVID-19 pandemic in general [12].

Our study was limited by the fact that it only concerns the Casablanca region, and only the patients that had access to our hospital, as many patients in our country do not have ease of access to health facilities. It was also limited by the fact that it only applies to the diagnosis and immediate treatment of patients, as the long-term outcome of our patients may be more heavily impacted by the initial staging of cancer than the short-term.

## 5 Conclusion

The COVID-19 pandemic had an undeniable impact on the staging of digestive cancer but had no effect on the short-term outcome of our patients and on their treatment. But the long-term effect of the pandemic on the survival of our patients remains to be evaluated and needs further studies in the future such as long-term cancer survival and survival without recurrence study.

This can help us better understand the real impact of the COVID-19 pandemic and avoid mistakes in the future, especially in light of the Sars-CoV-2 variants and the subsequent waves of the pandemic that may occur.

## Ethical approval

Ethical approval was provided by the board at university Mohamed 6 of health sciences.

## Source of funding

No funding was provided for this study.

## Author contributions

Mouni Omar: study design, concept data collection and analysis, writing the paper.

Idrissi Anas: data collection.

Bouziane Mohamed: data collection.

Ahid Samir: data analysis and writing the paper.

Sair Khalid: finale correction and approval

## Registration of research studies

1. Name of the registry: http://www.researchregistry.com
2. Unique Identifying number or registration ID: researchregistry8186
3. Hyperlink to your specific registration (must be publicly accessible and will be checked): https://www.researchregistry.com/register-now#home/registrationdetails/62f24bdf59b93d002147f4e7/

## Provenance and peer review

Not commissioned, externally peer-reviewed.

## Consent

Patient consent was obtained verbally and no patient identifying details are present in the study.

## Guarantor

The Guarantor is the one or more people who accept full responsibility for the work and/or the conduct of the study, had access to the data, and controlled the decision to publish.

## Declaration of competing interest

The authors declare no conflict of interest.
